# Effect of MnO_2_ Nanoparticles Stabilized with Cocamidopropyl Betaine on Germination and Development of Pea (*Pisum sativum* L.) Seedlings

**DOI:** 10.3390/nano14110959

**Published:** 2024-05-30

**Authors:** Andrey Nagdalian, Andrey Blinov, Alexey Gvozdenko, Alexey Golik, Zafar Rekhman, Igor Rzhepakovsky, Roman Kolesnikov, Svetlana Avanesyan, Anastasiya Blinova, Maxim Pirogov, Pavel Leontev, Alina Askerova, Evgeniy Tsykin, Mohammad Ali Shariati

**Affiliations:** 1 Laboratory of Food and Industrial Biotechnology, Faculty of Food Engineering and Biotechnology, North Caucasus Federal University, 355017 Stavropol, Russia; vikalinka04@mail.ru (A.A.);; 2 Department of Physics and Technology of Nanostructures and Materials, Physical and Technical Faculty, North Caucasus Federal University, 355017 Stavropol, Russia; avblinov@ncfu.ru (A.B.); agvozdenko@ncfu.ru (A.G.); abgolik@ncfu.ru (A.G.); zaarekhman@ncfu.ru (Z.R.); aablinova@ncfu.ru (A.B.); mpirogov@ncfu.ru (M.P.); psleontev@ncfu.ru (P.L.); 3 Interdepartmental Scientific and Educational Laboratory of Experimental Immunomorphology, Immunopathology and Immunobiotechnology, Faculty of Medicine and Biology, North Caucasus Federal University, 355017 Stavropol, Russia; irzhepakovskii@ncfu.ru (I.R.); savanesian@ncfu.ru (S.A.); 4 Scientific Department, Saints Petersburg State Agrarian University, 190005 Pushkin, Russia; roman-koles@bk.ru; 5 Semey Branch of Kazakh Research Institute of Processing and Food Industry, Almaty 050060, Kazakhstan

**Keywords:** manganese dioxide, nanotechnology, nano-priming, trace element, toxicity, ecotoxicology, fortification, bioconversion

## Abstract

This study aimed to synthesize, characterize, and evaluate the effect of cocamidopropyl betaine-stabilized MnO_2_ nanoparticles (NPs) on the germination and development of pea seedlings. The synthesized NPs manifested as aggregates ranging from 50–600 nm, comprising spherical particles sized between 19 to 50 nm. These particles exhibited partial crystallization, indicated by peaks at 2θ = 25.37, 37.62, 41.18, 49.41, 61.45, and 65.79°, characteristic of MnO_2_ with a tetragonal crystal lattice with a I4/m spatial group. Quantum chemical modelling showed that the stabilization process of MnO_2_ NPs with cocamidopropyl betaine is energetically advantageous (∆E > 1299.000 kcal/mol) and chemically stable, as confirmed by the positive chemical hardness values (0.023 ≤ η ≤ 0.053 eV). It was revealed that the interaction between the MnO_2_ molecule and cocamidopropyl betaine, facilitated by a secondary amino group (NH), is the most probable scenario. This ascertain is supported by the values of the difference in total energy (∆E = 1299.519 kcal/mol) and chemical hardness (η = 0.053 eV). These findings were further confirmed using FTIR spectroscopy. The effect of MnO_2_ NPs at various concentrations on the germination of pea seeds was found to be nonlinear and ambiguous. The investigation revealed that MnO_2_ NPs at a concentration of 0.1 mg/L resulted in the highest germination energy (91.25%), germinability (95.60%), and lengths of roots and seedlings among all experimental samples. However, an increase in the concentration of preparation led to a slight growth suppression (1–10 mg/L) and the pronounced inhibition of seedling and root development (100 mg/L). The analysis of antioxidant indicators and phytochemicals in pea seedlings indicated that only 100 mg/L MnO_2_ NPs have a negative effect on the content of soluble sugars, chlorophyll a/b, carotenoids, and phenols. Conversely, lower concentrations showed a stimulating effect on photosynthesis indicators. Nevertheless, MnO_2_ NPs at all concentrations generally decreased the antioxidant potential of pea seedlings, except for the ABTS parameter. Pea seedlings showed a notable capacity to absorb Mn, reaching levels of 586.5 μg/L at 10 mg/L and 892.6 μg/L at 100 mg/L MnO_2_ NPs, surpassing the toxic level for peas according to scientific literature. However, the most important result was the observed growth-stimulating activity at 0.1 mg/L MnO_2_ NPs stabilized with cocamidopropyl betaine, suggesting a promising avenue for further research.

## 1. Introduction

The field pea (*Pisum sativum* L.) is a leguminous crop of significant food and feed importance. With its relatively high yields and relatively low production costs, the pea has emerged as a key alternative protein source, driving its increasing demand over recent decades [1,2]. Global pea production surpassed 20 million tons, with a projected 7% growth by 2026 [3,4]. 

Pea plants exhibit adaptability to diverse soil and climatic conditions, facilitated by their relatively short growing season [5,6]. Despite its adaptability, pea cultivation faces challenges stemming from the crop’s high sensitivity to various abiotic stress factors. Fluctuations in yields not only jeopardize the sustainability of pea production, but also impedes market development [7,8,9,10]. It is noteworthy that modern, highly productive pea varieties have substantial metabolic requirements, necessitating adequate nutrient supply in various forms and application methods for both macro- and microelements [11]. Insufficient manganese (Mn) content in the soil frequently emerges as a significant limiting factor for pea growth and development [12,13,14]. Therefore, addressing this issue is crucial for optimizing pea cultivation and ensuring robust yields [15,16]. 

Mn is present in peas in very small quantities (14–15 mg/g), yet its role in growth, development and crop formation is indispensable [17]. This element actively participates in photosynthesis and various physiological processes, being a constituent of numerous ribosomes, chloroplasts, and enzymes [18]. In instances of manganese deficiency, chlorophyll synthesis is impaired, leading to the appearance of light yellow spots on leaves while the veins remain green [19]. Furthermore, according to Priyadarsini et al. [20], Mn has the highest number of significant relationships with other trace elements, implying that a manganese deficiency can trigger a broader mineral imbalance in peas. In the later phases of ontogenesis, signs of manganese deficiency can resemble those of iron deficiency. Severe deficiency can result in stunt growth, and, in extreme cases, the biomass gain may be negligible. Manganese deficiency is most commonly observed in alkaline and neutral soils rich in humus, as well as in cases of moisture deficiency [21]. Importantly, an Mn deficiency is not solely attributable to the absence or insufficient content of the element in soil. The bioavailability of Mn forms plays a crucial role, depending on factors such as shape, size, stability, and soil conditions (acidity, humidity, buffer properties) [22,23,24].

Currently, a considerable body of research has accumulated on the utilization of Mn forms in crop production. Among these forms of Mn studied, nanoscale forms emerge as the most promising due to their high activity and lower consumption [25]. It was discovered in a previous work that 0.05 mg/L Mn_x_O_y_ nanoparticles (NPs) increase the germination energy of barley seeds by 50%, while the seedling length increases by 68% when compared with the control [26]. Kasote et al. [27] reported that ≤40 mg/L Mn_2_O_3_ NPs significantly influenced the phenolic acid and phytohormone profiles of watermelon seedlings, leading to increased crop productivity. Ye et al. [28] found that Mn NPs were less phytotoxic when compared to other Mn forms and were more effective in minimizing abiotic stresses in plants. Nawaz et al. [29] demonstrated the positive nutritive impact of 15 mg/L MnO NPs, which increased the callus induction in *Moringa oleifera*. In a study on wheat biofortification, Dimkpa et al. [30] reported that Mn_2_O_3_ NPs were the most effective form, resulting in better seedling growth and yield. Pradhan et al. [31] studied the molecular, biochemical, and biophysical effects of Mn NPs on *Vigna radiate*, revealing the stimulation of photosynthesis processes. Additionally, Heena et al. [32] highlighted the highly beneficial effects of Mn NPs on the growth, yield, and economics of garden peas, confirming the potential of treating peas with Mn nanoforms. However, all discussed works showed a potential toxic effect of Mn NPs at higher concentrations, underscoring the need for further experimentation and careful consideration when applying Mn nanoforms to peas.

This study is distinctive in its use of an ampholytic surfactant, cocamidopropyl betaine, as a stabilizer, enabling the maintenance of nanoparticle (NP) stability at any pH, owing to the presence of positive and negative charges in the stabilizer molecule. Cocamidopropyl betaine was chosen as the stabilizer agent because of its amphiphilic properties and great performance in terms of stabilization of Se NPs in the previous work [33]. Consequently, the use of cocamidopropyl betaine as a stabilizer facilitates achieving the desired morphology and optimal particle size, while maintaining the functional properties of MnO_2_ NPs. Thus, considering the results of the literature review and our own results, the aim of this study concerns the synthesis, characterization, and study of the effect of MnO_2_ NPs stabilized with cocamidopropyl betaine on the germination and development of pea seedlings. 

## 2. Materials and Methods

### 2.1. Chemicals and Materials

In the present study, reagent-grade chemicals and grade A glassware were used. The conductivity of the distilled water used in this work was less than 2 µS/cm. The following chemicals were obtained from StavReaChem (Stavropol, Russia) and were used as received: potassium permanganate, L-methionine, cocamidopropyl betaine, and sodium hydroxide. Lenreactive (St. Petersburg, Russia) provided phloroglucinol, hydrochloric acid, ascorbic acid, sulfuric acid, gallic acid, sodium phosphate, sodium carbonate, iron chloride, phosphate buffer, and ethanol. Picric acid, potassium ferricyanide, trichloroacetic acid, ammonium molybdate, Tris-HCl buffer, chromogen, ABTS, trolox, and folin reagents were purchased from Sigma-Aldrich (St. Louis, MO, USA). Pea (*Pisum sativum* L.) seeds were purchased from the Timiryazevsky nursery of the Russian State Agrarian University-Moscow Timiryazev Agricultural Academy (Moscow, Russia) in August–October 2023. The selected seeds were not subjected to any prior additional processing.

### 2.2. Synthesis of MnO_2_ NPs 

The synthesis of MnO_2_ NPs involved mixing a 0.14 M aqueous solution of KMnO_4_ and 0.24 M aqueous solution of L-methionine in the presence of cocamidopropyl betaine with ω = 0.085%. The resulting sample underwent centrifugation 3 times at 3000 rpm using a Gyrozen 1580R centrifuge (Gyrozen, St. Petersburg, Russia). Subsequently, the sample was dried in a drying chamber IKA OVEN 125 basic dry (IKA-Werke GmbH & Co. KG, Staufen, Germany) at 65 °C for 24 h.

### 2.3. Characterization and Modelling of MnO_2_ NPs

Several methods were employed to characterize the produced MnO_2_ NPs. A micrograph of MnO_2_ NPs was obtained using an AZtecEnergy Standard X-max 20 system and a MIRA3-LMH scanning electron microscope (Tescan, Brno, Czech Republic) to determine its elemental composition. The phase composition of samples of MnO_2_ NPs was examined using an X-ray diffractometer Empyrean (PANalytical, Almelo, the Netherlands). The size of MnO_2_ NPs was analyzed using the dynamic light scattering (DLS) method with a Photocor Complex device (Antek-97, Moscow, Russia). The data obtained were processed using DynaLS v.2.0 software (Antek-97, Moscow, Russia). For DLS analysis, MnO_2_ NP samples were diluted four times with distilled water. Furthermore, the ζ-potential was investigated using acoustic and electroacoustic spectroscopy with a DT-1202 device (Dispersion Technology Inc., Bedford Hills, NY, USA).

Quantum chemical modeling was used to investigate the interaction of a MnO_2_ molecule with cocamidopropyl betaine through several functional groups (secondary amino group, carboxyl group, and nitrogen) to assess the viability of cocamidopropyl betaine as a stabilizer of MnO_2_ NPs. The stabilization process of CuO NPs with stabilizers was also studied using QChem v. 6.2 software (QChem, Pleasanton, CA, USA) with a IQmol molecular editor (QChem, Pleasanton, CA, USA). 

Calculations were performed at the data processing center (Schneider Electric, Moscow, Russia) of the North Caucasus Federal University (Stavropol, Russia). Calculations were carried out with the following parameters: calculation—energy, method—B3LYP, basis—6-31G*, convergence—5, force field—Chemical. The total energy of the molecular complex (E), the energy of the most occupied molecular orbital (E_HOMO_), and the energy of the least occupied molecular orbital (E_LUMO_) were considered as quantum chemical variables. Based on the calculated values, the total energy difference (∆E) of cocamidopropyl betaine and the MnO_2_–cocamidopropyl betaine molecular complex, and the chemical rigidity (η) of the molecular system equal to half of the difference between E_HOMO_ and E_LUMO_ were obtained.

To confirm the results of the quantum chemical modeling, IR spectra of cocamidopropyl betaine, MnO_2_ NPs, and the MnO_2_–cocamidopropyl betaine molecular complex were acquired and analyzed using an FSM-1201 FTIR spectrometer (Infraspek, St. Petersburg, Russia). Measurements were recorded in the range 500–4000 cm^−1^.

### 2.4. Pea Seed Preparation and Investigation

Pea seeds (75 units per group) were evenly distributed in Petri dishes, with each dish containing 25 units placed on filter paper under optimal humidification conditions at a temperature of 20 °C for 7 days. The ratio of liquid phase to seeds was maintained at 4:5. Considering the results of the literature review, the liquid phase consisted of the following: distilled water (control group), 0.1 mg/L MnO_2_ NPs solution (experimental group 1), 1 mg/L MnO_2_ NPs solution (experimental group 2), 10 mg/L MnO_2_ NPs solution (experimental group 3), and 100 mg/L MnO_2_ NPs solution (experimental group 4). The germination energy, germinability, and the linear dimensions of seedlings and roots were evaluated according to the ISTA (2006) standard every 3 days for the 9 days of germination. Further investigation was focused on pea seedlings considering potential microgreens applications.

### 2.5. Investigation of Pea Seedlings 

#### 2.5.1. Energy-Dispersive X-ray Spectroscopy

Seedling cut micrographs with Mn mapping were obtained using a scanning electron microscope MIRA3-LMH (Tescan, Brno-Kohoutovice, Czech Republic) with a system for determining the elemental composition AZtecEnergy Standard/X-max 20 (standard). Seedling samples were freeze-dried at −40 °C. A double-sided conductive carbon tape was glued on a standard instrumentation table (12 mm), onto which the freeze-dried seedling samples were applied. Finally, a carbon coating with a thickness of about 10 nm was deposited. The parameters of the measurement were as follows: Voltage: 10 kV, Work distance: 4.9 mm, and an In-Beam secondary electron detector.

Micrographs of pea seedlings with manganese mapping were acquired using a scanning electron microscope (SEM) model MIRA3-LMH (Tescan, Brno-Kohoutovice, Czech Republic), equipped with an AZtecEnergy Standard/X-max 20 system for determining the elemental composition. Seedling samples underwent freeze-drying at −40 °C. Double-sided conductive carbon tape (12 mm) was applied to a standard instrumentation table, onto which the freeze-dried seedling samples were applied. Subsequently, a carbon coating approximately 10 nm thick was deposited. The measurement parameters were set as follows: Voltage: 10 kV, Working distance: 4.9 mm, and an In-Beam secondary electron detector.

#### 2.5.2. Antioxidant Indicators and Phytochemicals in Pea Seedlings

For probe preparation, 100 mg of freeze-dried pea seedlings were grounded and soaked in 10 mL of 70% ethanol. The extracts underwent centrifugation at 13,000 rpm for 15 min using MicroCL 17R centrifuge (Thermo FS, Waltham, MA, USA) to investigate the supernatants. All parameters studied, except for the dry matter value and Mn content, were recalculated per g of freeze-dried sample.

- Dry matter

The dry matter was determined in fresh seedlings using an Ohaus MB 25 (Ohaus Corporation, Parsippany, NJ, USA) moisture meter at 105 °C.

- Soluble sugars

Soluble sugars in the extract were determined via the reaction with picric acid using an SF 102 UV spectrophotometer (Aquilon, Moscow, Russia) according to Mukherjee et al. [34]. Soluble sugars were expressed in mg of glucose per g of freeze-dried seedlings (mg G/g).

- Ferric reducing antioxidant power (FRAP)

The FRAP assay was carried out following the methods outlined by Hazra et al. [35] and Moualek et al. [36] with slight modifications. Specifically, 25 µL of extract was mixed with 2 mL of phosphate buffer (pH 6.6) and 1 mL of 1% potassium ferricyanide. This mixture was then incubated in a climatic chamber (Thermo FS, Waltham, MA, USA) at 50 °C for 20 min. The reaction was terminated by the addition of 1 mL of 10% trichloroacetic acid. Subsequently, the mixture was centrifuged at 3000 rpm g for 10 min using MicroCL 17R centrifuge (Thermo FS, Waltham, MA, USA). Next, 1 mL of freshly prepared 0.1% FeCl_3_ was introduced to the supernatant, and the absorption was measured at λ = 700 nm using the SF 102 UV spectrophotometer (Aquilon, Moscow, Russia). Ascorbic acid was served as a standard. The FRAP was expressed in mg of ascorbic acid equivalent per g of freeze-dried seedings (mg AAE/g).

- Total antioxidant activity (TAA)

The TAA was assayed with the use of the phospho-molybdenum method [37,38]. To begin, 25 µL of extract was mixed with 4 mL of the reagent solution (0.6 M sulfuric acid, 28 mM sodium phosphate, and 4 mM ammonium molybdate). The mixture was then incubated in a climatic chamber (Thermo FS, Waltham, MA, USA) at 95 °C for 90 min, followed by being cooled to room temperature. Subsequently, the absorbance was measured at λ = 695 nm using the SF 102 UV spectrophotometer (Aquilon, Moscow, Russia). Ascorbic acid was used as a standard. The TAA was expressed in mg of ascorbic acid equivalent per g of freeze-dried seedings (mg AAE/g).

- ABTS radical scavenging activity

ABTS (2,2′-azino-bis(3-ethylbenzothiazoline-6-sulfonic acid)) radical scavenging activity was determined according to the procedure described by Liu et al. [39] with slight modifications. Initially, a working solution was prepared by dissolving the chromogen containing ABTS^+^ in 20 mL of Tris-HCl buffer. The extract (0.030 mL) was added to 2.97 mL of the ABTC solution and allowed to stand for 3 min. Next, the optical density of resulting mixture was measured at λ = 734 nm using the SF 102 UV spectrophotometer (Aquilon, Moscow, Russia). Trolox was used as a standard. In the negative control, 70% ethanol was used instead of the extract. ABTS radical scavenging activity was expressed in μM of trolox equivalent per g of freeze-dried seedings (μM TE/g).

- Total phenolics

Phenols were quantified using the Folin–Ciocalteu method [40]. Firstly, Folin reagent (diluted 10 times) was added to 0.2 mL of the extract. Subsequently, 0.5 mL of 20% Na_2_CO_3_ and 2.5 mL of distilled water were added, and the resulting solution was allowed to stand for 45 min at room temperature in a dark place. The optical density was then measured at λ = 760 nm using the SF 102 UV spectrophotometer (Aquilon, Moscow, Russia). Gallic acid served as the standard reference. The content of the phenolic compounds was expressed in mg of gallic acid equivalent per g of freeze-dried seedlings (mg GAE/g).

- Chlorophyll a, Chlorophyll b, and Total carotenoids content

The content of chlorophyll a, chlorophyll b, and total carotenoids was measured spectrophotometrically. The extract was mixed with 95% ethanol and then clarified via centrifugation at 10,000 rpm for 15 min at 4 °C using MicroCL 17R centrifuge (Thermo FS, Waltham, MA, USA). The absorbance of the supernatant was measured using the SF 102 UV spectrophotometer (Aquilon, Moscow, Russia), and the concentrations of chlorophyll a, chlorophyll b, and carotenoids were calculated considering 95% ethanol, as described by Alam et al. [41].

- Mn content

Mn content in pea seedlings was determined using the atomic absorption spectrometer MGA-1000 (Lumex, St. Petersburg, Russia). 

### 2.6. Statistical Data Processing

The experiments were carried out in threefold biological and fivefold analytical repetitions. All parameters obtained underwent one-way analysis of variance (ANOVA) and Student’s *t*-test (*p*  <  0.05) using the statistical package STATISTICA v. 12 for Windows (Statsoft, Tulsa, Oklahoma, USA). Data regarding the root and seedling length were statistically processed using Python 3.10 software with the Jupyter Notebook web-based interactive computing platform, using the pandas, numpy, sklearn, matplotlib, and seaborn libraries (Source). Microsoft Excel 2010 and Origin v. 9.0 software were also employed for histogram and graph creation based on the results of the data processing.

## 3. Results and Discussion

### 3.1. Characterization of MnO_2_ NPs Stabilized with Cocamidopropyl Betaine

**Figure 1:**
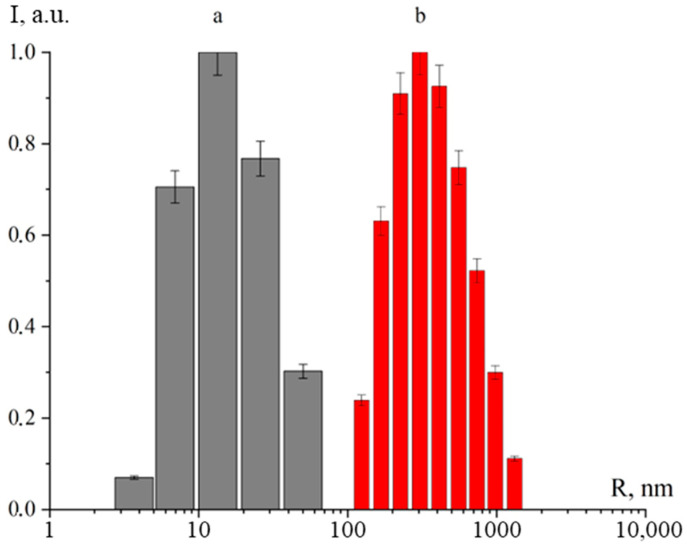
Histograms of the distribution of the average hydrodynamic radius (R) of MnO_2_ NPs with a stabilizer (**a**) and without a stabilizer (**b**).

At the first stage, the average hydrodynamic radius and ζ-potential of MnO_2_ NPs were studied to validate the effectiveness of cocamidopropyl betaine as a stabilizer. The outcomes of DLS are presented in Figure 1.

DLS showed that the average hydrodynamic radius of MnO_2_ NPs stabilized with cocamidopropyl betaine (36 nm) is significantly lower than the size of MnO_2_ NPs without a stabilizer (420 nm). At the same time, results from acoustic and electroacoustic spectroscopy indicate that the ζ-potential of the stabilized sample (+32.64 mV) was higher than in the non-stabilized sample (+1.34 mV). These findings conclusively demonstrate the effectiveness of using cocamidopropyl betaine as a stabilizer.

**Figure 2:**
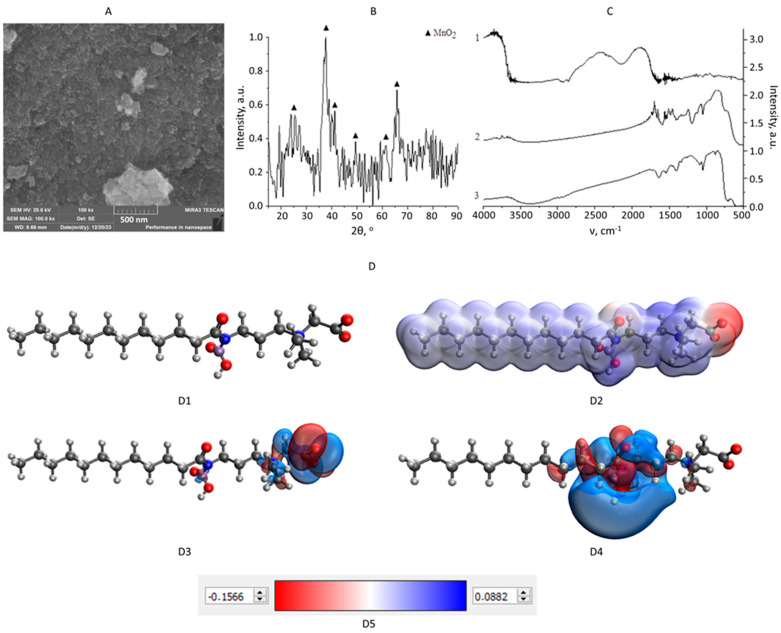
Results of the characterization of MnO_2_ NPs stabilized with cocamidopropyl betaine: scanning electron microscopy at magnification of ×100,000 (**A**); X-ray diffractogram (**B**); FTIR spectra (**C**): cocamidopropyl betaine (1), MnO_2_ NPs stabilized with cocamidopropyl betaine (2), MnO_2_ NPs (3); Quantum chemical modelling of interactions of MnO_2_ NPs with cocamidopropyl betaine molecule through secondary amino group (**D**): model of a molecular complex (**D1**), electron density distribution (**D2**), the highest occupied molecular orbital HOMO (**D3**), the lowest unoccupied molecular orbital LUMO (**D4**), the gradient of the electron density distribution (**D5**).

The results of the characterization of MnO_2_ NPs stabilized with cocamidopropyl betaine are depicted in Figure 2. The analysis of SEM micrographs (Figure 2A) revealed that synthesized MnO_2_ NPs are aggregated structures measuring 50–600 nm, composed of spherical particles of 19 to 50 nm. The study of the phase composition (Figure 2B) showed that the stabilization of Mn_x_O_y_ NPs with cocamidopropyl betaine leads to the formation of amorphous MnO_2_ [42,43]. Further analysis of the obtained diffractogram showed the partial crystallization of the sample, evidenced by maxima at 2θ = 25.37, 37.62, 41.18, 49.41, 61.45, and 65.79°, characteristic of MnO_2_ with a tetragonal crystal lattice with a spatial group I4/m [44]. 

The stabilization process of MnO_2_ NPs with cocamidopropyl betaine was studied using quantum chemical modelling. As a result, models of molecule, electron density distribution, HOMO, and LUMO were obtained and presented in Figure 2D and Supplementary Figures S1–S3. Additionally, quantum chemical calculations were also carried out and presented in Table 1. 

According to Table 1, the stabilization process of MnO_2_ NPs with cocamidopropyl betaine is energetically favorable (∆E > 1299.000 kcal/mol) and chemically stable, as indicated by the positive chemical hardness values (0.023 ≤ η ≤ 0.053 eV) [45]. It was revealed that the interaction of the MnO_2_ molecule with cocamidopropyl betaine via a secondary amino group (NH) is the most probable, as evidenced by the values of the difference in total energy (∆E = 1299.519 kcal/mol) and chemical hardness (η = 0.053 eV). The electron density distribution model (Figure 2(D2)) indicates a transfer of positive charge from the hydrocarbon radical (CH) towards the MnO_2_ molecule. Additionally, there is a shift of the LUMO towards the MnO_2_ molecule, confirming the chemical interaction between MnO_2_ and cocamidopropyl betaine [46]. When the MnO_2_ molecule interacts with cocamidopropyl betaine through the carboxylate anion (COO^−^), the ∆E value increases to 1299.622 kcal/mol, indicating a high energy benefit of the process [47]. 

Meanwhile, the η value drops to 0.023 eV, indicating the relatively low chemical stability of the interaction [45]. In this case, the oxygen atoms in the MnO_2_ molecule acquire a negative charge [48]. Conversely, as seen from Table 1, the interaction of the MnO_2_ molecule with cocamidopropyl betaine through an ionized amino group is chemically stable (η = 0.053 eV), albeit less energetically advantageous (∆E = 1299.107 kcal/mol). Consequently, the MnO_2_ molecule acquires a positive charge [48].

The results of quantum chemical modelling were confirmed using FTIR spectroscopy. The obtain spectra are shown in Figure 2C, and their description is presented in Table 2. 

According to Table 2, a significant decrease in intensity is observed, ranging from 1591 to 1558 cm^−1^, corresponding to the fluctuations of the NH group [49]. This finding confirms the results of quantum chemical analysis, which demonstrates the interaction between the MnO_2_ molecule and the secondary amino group of cocamidopropyl betaine. Consequently, the results justify the utilization of cocamidopropyl betaine as a stabilizing agent for MnO_2_ NPs.

### 3.2. Effect of MnO_2_ NPs Stabilized with Cocamidopropyl Betaine on Germination of Pea Seeds

The effect of MnO_2_ NPs at various concentrations on the germination of pea seeds was found to be nonlinear and ambiguous. The research results revealed that seeds from the first experimental group (0.1 mg/L) exhibited the highest germination energy (91.25%). In contrast, the percentage germination of pea seeds from the fourth experimental group (100 mg/L) was the lowest (86.33%) when compared to other groups. Samples from the second group (1 mg/L) and the third group (10 mg/L) showed average values of germination energy (90.87% and 90.10%, respectively), albeit this was slightly higher than in the control group (87.45%). Ensuring the development of healthy, fully formed seedlings is a crucial prerequisite for achieving high yields of pea seeds [50]. 

The highest germinability value, 95.60%, was recorded following treatment with 0.1 mg/L MnO_2_ NPs, which is 8.8% higher than seeds germinated in distilled water (87.77%). Consistent with the results of germination energy, germinability in the second group (1 mg/L) and the third group (10 mg/L) surpassed that of the control group, reaching 93.33% and 91.10%, respectively. However, it was discovered that 100 mg/L MnO_2_ NPs negatively affected the germinability of pea seeds, resulting in 83.33%, equivalent to pea seeds stored for 5 years with reduced morphofunctional properties [51]. 

Thus, the results obtained revealed the growth stimulation effect of MnO_2_ NPs at concentrations of 0.1–10 mg/L. The inhibitory effect of 100 mg/L MnO_2_ NPs can be associated with the potential toxic effect on pea seeds at higher concentrations, which is in line with the results of our previous work [26] and the reports of another researcher [27,29,30,52,53,54].

**Figure 3:**
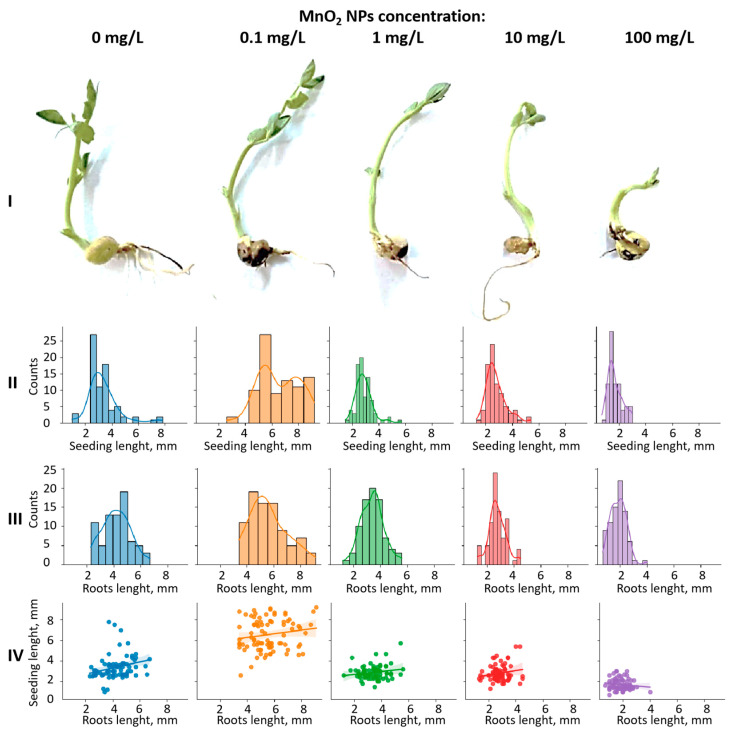
Effect of MnO_2_ NPs on germination of pea seeds: profile view of experimental samples at the same scale (**I**); distribution of seedling length depending on concentration of MnO_2_ NPs (**II**); distribution of roots length depending on concentration of MnO_2_ NPs (**III**); seedling length: roots length ratio at different concentrations of MnO_2_ NPs (**IV**).

The same trend was observed when evaluating the effect of MnO_2_ NPs on the seedling and root length (Figure 3). Statistical data processing (Supplementary, Tables S1–S3, Figures S4–S7) showed a significant difference in the length of roots and seedlings as a function of the MnO_2_ NP concentration. The highest growth simulation was revealed following treatment with 0.1 mg/L MnO_2_ NPs. Increasing the concentration of preparation led to a slight suppression of growth (1–10 mg/L) and a pronounced inhibition of seedling and root development (100 mg/L) when compared to the control. These results correspond to those of Pradhan et al. [31] and confirm the prospect of using MnO_2_ NPs at low concentrations as a growth stimulator for pea seeds.

### 3.3. Effect of MnO_2_ NPs Stabilized with Cocamidopropyl Betaine on Parameters of Pea Seedlings

**Figure 4:**
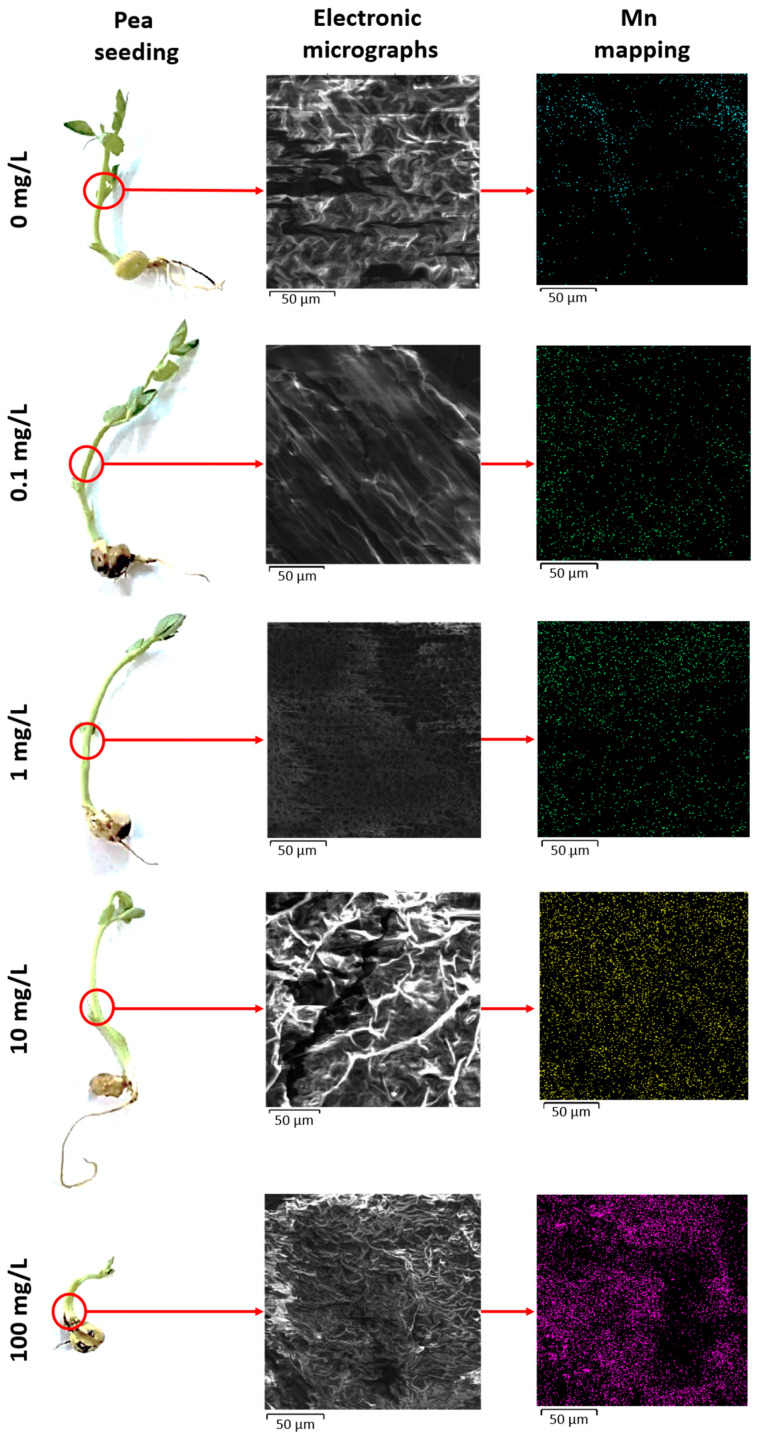
Energy-dispersive X-ray spectroscopy of experimental pea seedlings.

Mn is crucial for various physiological and biochemical processes in plants. However, excessive concentrations of Mn can lead to phytotoxicity, as evidenced by reduced seed germinability, germination energy, and impaired seedling and root growth, as discussed in the literature review. High Mn levels can induce oxidative stress in plants by generating ROS, leading to lipid peroxidation, disrupting antioxidant enzymes, reducing photosynthesis and metabolism parameters, interfering with the uptake, and even damaging cell structure and modulating the gene expression [55,56,57,58]. Visually, Mn toxicity may result in inhibited root and seedling growth and, in severe cases, plant death [59,60]. Understanding the biosorption capacity of pea seeds and the mechanisms of the translocation and accumulation of MnO_2_ nanoparticles is essential for this study. Energy-dispersive X-ray spectroscopy of experimental pea seedlings was conducted, and the resulting electronic micrographs and Mn mapping are shown in Figure 4. 

The release of Mn^4+^ from MnO_2_ NPs leads to the Mn^4+^ bioaccumulation in pea seeds and its distribution in roots and seedlings. Generally, plants can absorb and translocate NPs into various tissues, with roots being the primary target for absorption and accumulation [61]. For instance, Ahmed et al. [62] observed that, at a concentration of 2 mg/L, CuO NPs exhibited intense translocation from roots to seedlings and leaves of maize, leading to the higher bioaccumulation of Cu in aboveground tissues when compared to roots. Similar observations were reported for rice roots and seedlings by Peng et al. [63].

During the translocation from roots to seedlings and leaves, metal NPs may form complexes with cysteine, citrate, and phosphate ligands. Additionally, Mn can be translocated from roots to seedlings via xylem and re-translocated to roots via the phloem, likely involving transformation Mn^2+^-Mn^4+^-Mn^7+^ [23,64,65]. At optimal concentrations, Mn activates photosynthesis processes and positively effects seed germination [31]. The residual Mn is normally immobilized in the cytosol through chelation and sequestration with Mn-related proteins and stores in vacuoles [66,67]. 

This mechanism sheds insights onto why 0.1 mg/L MnO_2_ NPs stimulated the growth of pea seedlings and resulted in the highest germination energy and germinability of seeds. According to the results obtained, MnO_2_ NPs at concentrations of 100 mg/L disrupt the natural mechanism of Mn assimilation and utilization, leading to phytopathological consequences, as depicted in Figure 4. Mn mapping obtained via energy dispersive X-ray spectroscopy showed the significant redistribution of the Mn atomic presence. The increase in the MnO_2_ NP concentration led to intense Mn accumulation. The widespread distribution of Mn in seedlings treated with 100 mg/L MnO_2_ NPs indicates a high biosorption capacity of pea seedlings, consistent with similar results reported by Kasote et al. [27] in watermelon seedlings. However, this led to a reduction in germinability and germination energy in pea seeds and inhibited the growth of their seedlings and roots.

The high content of Mn, as a redox active element, can lead to active ROS generation in pea seedlings through interactions with the photosynthetic electron transport system [30,68,69]. Considering the literature data and the results of energy-dispersive X-ray spectroscopy, experimental samples were studied for antioxidant indicators and phytochemicals content (Table 3). 

According to Table 3, MnO_2_ NPs led to a reduction in the water content in seedlings with an increase in the concentration. Margenot et al. [70] attributed this decrease in water content to the negative effect of high concentrations of metal oxide NPs on root function and water transport. It is important to note that ROS generating at MnO_2_ NPs treatment can induce oxidative stress, leading to multifactor damages and cell death [71,72]. Plants have adopted strategies to counteract these toxic effects through both antioxidant enzymatic and non-enzymatic activity [73,74].

Thus, to assess the effect of MnO_2_ NPs on the antioxidant system in pea seedlings, the FRAP, TAA, and ABTS were evaluated. It was found that the FRAP decreased by 2–3 times with increasing MnO_2_ NP concentrations, reaching its minimum value (21.16 mg AAE/g) at 100 mg/L MnO_2_ NPs. Similarly, MnO_2_ NPs led to a decrease in the TAA in pea seedlings, ranging from 23% (0.1 mg/L) to 53% (100 mg/L), indicating a significant depletion of total antioxidant potential in the experimental samples.

Surprisingly, the ABTS test revealed an inverse effect; the maximum value (24.7 μM TE/g) was detected at 0.1 mg/L MnO_2_ NPs, while other groups, including the control, had no statistically significant difference (21.4–23.1 μM TE/g). This observed trend might be attributed to the oxidative stress response and the activation of antioxidant enzymes [75,76]. 

The analysis of soluble sugars, chlorophyll a/b, and carotenoids revealed a single trend. It was found that MnO_2_ NPs at concentrations of 0.1–10 mg/L had a positive effect on the content of soluble sugars, chlorophyll a/b, and carotenoids in pea seedlings. These results align with the findings of Heena et al. [32], suggesting that this effect may be attributed to the stimulation of photosynthesis processes. However, at a concentration of 100 mg/L, MnO_2_ NPs likely disrupted the biochemical homeostasis in peas, leading to a significant decrease in the studied indicators of the photosynthesis intensity, which is consistent with findings of Pradhan et al. [31]. Nevertheless, it is noteworthy that a positive effect on photosynthesis processes was still observed at 10 mg/L MnO_2_ NPs, corresponding to results of Kasote et al. [27], Ye et al. [28], Nawaz et al. [29], and Dimpka et al. [30]. These studies, conducted with different crops and various forms of Mn_x_O_y_ NPs, found an upper optimal concentration range of 15–40 mg/L. 

Interestingly, the phenols content correlates well with carotenoids and chlorophyll a/b contents. Seedlings treated with 0.1, 1, and 10 mg/L MnO_2_ NPs had higher phenol contents (9.66, 10.68, and 9.71 mg GAE/g, respectively) than the control samples (9.53 mg GAE/g). Moreover, this indicator was even higher in samples from the second and third experimental groups. As expected, seeds treated with 100 mg/L MnO_2_ NPs contained only 8.75 GAE/g phenols in seedlings, representing an 8.2% decrease when compared to the control group. These results align with those of Ramesh et al. [77]. However, further studies on phenolic compounds are warranted to fully understand the nature of the observed effect. This is particularly critical as phenolic compounds may play a significant role in antioxidant capacities under Mn toxicity, as logically inferred from the results presented in Table 3.

The analysis of the Mn content in pea seedlings provided data that objectively supplemented the Mn mapping micrographs obtained via energy-dispersive X-ray spectroscopy. As mentioned earlier, pea seedlings exhibited a high absorbance capacity for Mn, reaching 892.6 μg/L at 100 mg/L MnO_2_ NPs. According to Donchaeva et al. [56], Mn inhibits pea growth and development in a dose- and time-dependent manner at accumulation concentrations ≥ 500 μg/L. Thus, experimental pea seedlings treated with 10 mg/L MnO_2_ NPs were also above the toxic level of Mn content (586.5 μg/L). 

Thus, it can be inferred that 100 mg/L MnO_2_ NPs stabilized with cocamidopropyl betaine have a pronounced toxic effect on pea seeds. This effect is also observed at concentrations of 10 mg/L, albeit to a lesser extent and not across all indicators. However, the most important result was the revealed growth-stimulating activity of 0.1 mg/L MnO_2_ NPs stabilized with cocamidopropyl betaine. This discovery opens up a new avenue of research into the application of MnO_2_ NPs as a stable bioavailable molecular complex based on essential trace elements (Mn), with a growth-promoting effect in the early germination stages of agricultural crops.

## 4. Conclusions

Within the scope of this study, manganese dioxide nanoparticles stabilized with cocamidopropyl betaine were synthesized. These nanoparticles formed aggregates ranging from 50–600 nm, composed of spherical particles between 19 and 50 nm with partial crystallization. The presence of maxima at 2θ = 25.37, 37.62, 41.18, 49.41, 61.45, and 65.79° indicates characteristic features of MnO_2_ with a tetragonal crystal lattice and spatial group I4/m. Quantum chemical modelling elucidated the optimal formation pathway of the MnO_2_ NPs–cocamidopropyl betaine molecular complex through a secondary amino group. This was confirmed by the values of the difference in total energy (∆E = 1299.519 kcal/mol) and chemical hardness (η = 0.053 eV), which were further validated using FTIR spectroscopy.

The effect of MnO_2_ NPs at various concentrations on the germination of pea seeds was found to be nonlinear and ambiguous. Notably, 0.1 mg/L MnO_2_ NPs provided the highest germination energy (91.25%), germinability (95.60%), and root and seedling lengths among all experimental samples. However, an increase in the concentration of the preparation led to a slight growth suppression (1–10 mg/L) and the pronounced inhibition of seedling and root development (100 mg/L). The analysis of antioxidant indicators and phytochemicals in pea seedlings showed that only 100 mg/L MnO_2_ NPs have a negative effect on the content of soluble sugars, chlorophyll a/b, carotenoids, and phenols. Conversely, lower concentrations showed a stimulating effect on photosynthesis indicators. 

However, MnO_2_ NPs at all concentrations generally decreased the antioxidant potential of pea seedlings, except for the ABTS parameter. Pea seedlings showed a high absorbance capacity to Mn, reaching 586.5 μg/L at 10 mg/L and 892.6 μg/L at 100 mg/L MnO_2_ NPs, thus surpassing the toxic threshold for peas according to the scientific literature.

The most important result was the revealed growth-stimulating activity of 0.1 mg/L MnO_2_ NPs stabilized with cocamidopropyl betaine. This discovery paves the way for a new direction of research into the application of MnO_2_ NPs as an essential trace element (Mn)-based bioavailable stable molecular complex with a growth stimulating effect in the early stages of agricultural crop germination.

## Figures and Tables

**Table 1:** Quantum chemical calculations of the stabilization process of MnO_2_ NPs with cocamidopropyl betaine.

Interaction	E, kcal/mol	∆E, kcal/mol	E_HOMO_, eV	E_LUMO_, eV	η, eV
	−1082.031	–	−0.150	0.016	0.083
Through the secondary amino group	−2381.138	1299.519	−0.154	−0.048	0.053
Through the carboxylate anion	−2381.653	1299.622	−0.034	0.011	0.023
Through the ionized amino group	−2381.550	1299.107	−0.143	−0.038	0.053

**Table 2:** Description of FTIR spectra.

MnO_2_ NPs		Cocamidopropyl Betaine		MnO_2_ NPs Stabilized with Cocamidopropyl Betaine	
*ν*, cm^−1^	Bond	*ν*, cm^−1^	Bond	*ν*, cm^−1^	Bond
607	*(Mn-O)*	565.00	*δ_oop_ (O–H*)	560.00	*(Mn-O)*
711	*(Mn-O)*	667.00	*δ_oop_ (O–H*)	1048.00	flexural *(O-H)*
879	*(Mn-O)*	713.00	*δ (NH*)	1198.00	*ν (C-N*)
1047	flexural *(O-H)*	896.00	*δ (CH_3_*)	1324.00	*ν (C-N*)
1089	flexural *(O-H)*	985.00	*δ (CH_3_*)	1408.00	*ν (CH_2_*) *δ (CH_2_*)
1402	*(O-H)*	1051.00	*ν (C-N*)	1481.00	*δ (CH_3_*)
1539	flexural *(O-H)*	1116.00	*ν (C-N*)	1547.00	flexural *(O-H)*
1643	*(O-H)*	1151.00	*ν (C-N*)	1569.00	*δ (NH*)
–	–	1192.00	*ν (C-N*)	1591.00	*δ (NH*)
–	–	1336.00	*ν (C-N*)	1664.00	*(O-H)*
–	–	1417.00	*ν (CH_2_*) *δ (CH_2_*)	–	–
–	–	1456.00	*δ (CH_3_*)	–	–
–	–	1469.00	*δ (CH_3_*)	–	–
–	–	1558.00	*δ (NH*)	–	–
–	–	1651.00	absorptive *(C=O)*	–	–
–	–	2152.00	absorptive *(C=O)*	–	–
–	–	2854.00	*ν (CH*) *δ (CH*)	–	–
–	–	2922.00	*ν (CH*) *δ (CH*)	–	–
–	–	2958.00	*(C-H)*	–	–

**Table 3:** Antioxidant indicators and phytochemicals in pea seedlings, *p* < 0.05.

Index	Concentration of MnO_2_ NPs, mg/L				
	0	0.1	1	10	100
Dry matter, %	13.27 ± 0.34	12.62 ± 0.35	13.00 ± 0.44	13.66 ± 0.39	15.46 ± 0.69
FRAP, mg AAE/g	61.12 ± 0.71	30.03 ± 0.65	31.91 ± 0.47	28.99 ± 0.34	21.16 ± 0.40
TAA, mg AAE/g	161.1 ± 1.4	123.2 ± 2.0	90.3 ± 2.8	90.0 ± 1.6	75.5 ± 2.7
ABTS, μM TE/g	23.1 ± 0.7	24.7 ± 0.8	23.0 ± 0.6	21.4 ± 0.8	22.2 ± 0.7
Soluble sugars, mg G/g	136.6 ± 4.1	148.3 ± 5.5	141.0 ± 3.2	127.3 ± 5.4	96 ± 2.9
Chlorophyll a, mg/g	0.53 ± 0.06	0.61 ± 0.05	0.72 ± 0.06	0.55 ± 0.05	0.35 ± 0.04
Chlorophyll b, mg/g	0.26 ± 0.02	0.30 ± 0.02	0.33 ± 0.03	0.26 ± 0.01	0.11 ± 0.01
Carotenoids, mg/g	0.11 ± 0.01	0.13 ± 0.01	0.15 ± 0.01	0.11 ± 0.01	0.08 ± 0.01
Phenols, mg GAE/g	9.53 ± 0.04	9.66 ± 0.03	10.68 ± 0.05	9.71 ± 0.04	8.75 ± 0.06
Mn content, μg/L	244.2 ± 1.2	256.9 ± 2.9	291.0 ± 4.1	586.5 ± 8.3	892.6 ± 14.2

FRAP—Ferric reducing antioxidant power; TAA—Total antioxidant activity; ABTS—2,2′-azino-bis(3-ethylbenzothiazoline-6-sulfonic acid).

## Data Availability

All raw data are available upon request from the corresponding author.

## Supplementary Materials

The following supporting information can be downloaded at: https://www.mdpi.com/article/10.3390/nano14110959/s1, Figure S1: Quantum chemical modelling of cocamidopropyl betaine molecule; Figure S2: Quantum chemical modelling of interaction of MnO_2_ NPs with cocamidopropyl betaine through carboxilat-anoin; Figure S3: Quantum chemical modelling of interaction of MnO_2_ NPs with cocamidopropyl betaine through the ionized amino group; Figure S4: Correlation heat map between log of MnO_2_ NPs concentration, root length and seedling (sprout) length; Figure S5: Box plot of dependence of roots length on MnO_2_ NPs concentration; Figure S6: Box plot of dependence of seedling (sprout) length on MnO_2_ NPs concentration; Figure S7: Distribution of length of roots and seedling; Table S1: ANOVA of dependence of roots length on concentration of MnO_2_ NPs; Table S2: ANOVA of dependence of seeding length on concentration of MnO_2_ NPs; Table S3: Statistically data processing.
