# Supplementary material for: Effect of MnO2 Nanoparticles Stabilized with Cocamidopropyl Betaine on Germination and Development of Pea (Pisum sativum L.) Seedlings
Source: Nanomaterials (Basel). 2024 May 30;14(11):959. doi: 10.3390/nano14110959 (PMC11174102; doi:10.3390/nano14110959)
Supplement: Supplementary file 1 [file nanomaterials-14-00959-s001.zip › nanomaterials-2992323-supplementary.pdf]

Supplementary

S1. Quantum chemical modelling

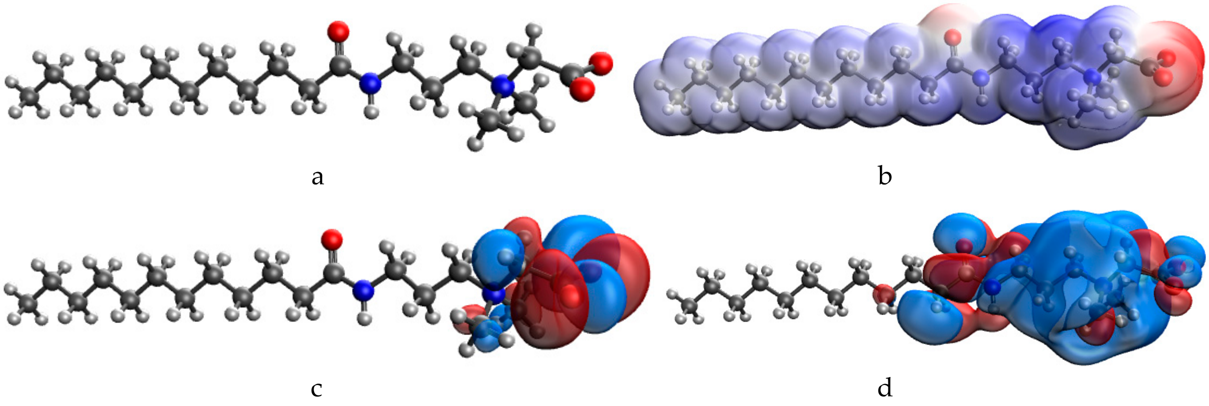

Figure S1. Quantum chemical modelling of cocamidopropyl betaine molecule: a) model of molecule; b) distribution of electron density; c) the highest occupied molecular orbital (HOMO); d) the lowest unoccupied molecular orbital (LUMO)

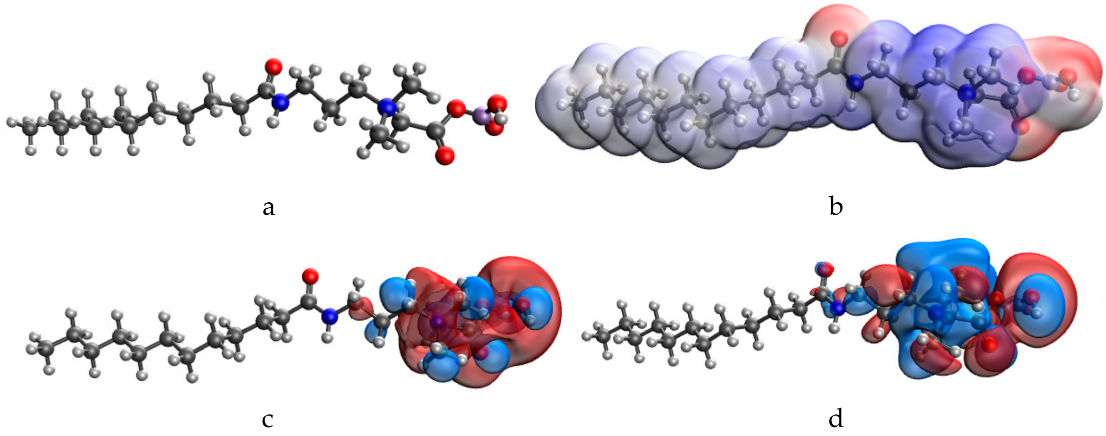

Figure S2. Quantum chemical modelling of interaction of MnO_2 NPs with cocamidopropyl betaine through carboxilat-anoin: a) model of molecular complex; b) distribution of electron density; c) the highest occupied molecular orbital (HOMO); d) the lowest unoccupied molecular orbital (LUMO)

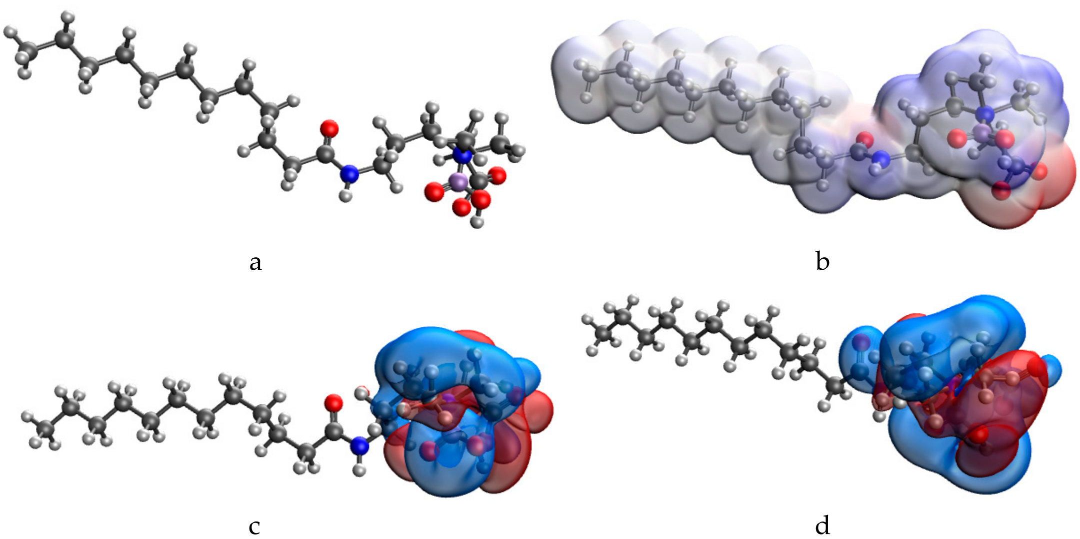

Figure S3. Quantum chemical modelling of interaction of MnO_2 NPs with cocamidopropyl betaine through the ionized amino group: a) model of molecular complex; b) distribution of electron density; c) the highest occupied molecular orbital (HOMO); d) the lowest unoccupied molecular orbital (LUMO)

S2. Details for statistically data processing

Table S1. ANOVA of dependence of roots length on concentration of MnO_2 NPs.

Index	Sum_sq	dF	F	PR (>F)
Solution	322.306	1.0	118.651	1.136e-24
Residual	1216.949	448.0	NaN	NaN

Table S2. ANOVA of dependence of seedling length on concentration of MnO_2 NPs.

Index	Sum_sq	dF	F	PR (>F)
Solution	346.089	1.0	92.506	4.941e-29
Residual	1676.094	448.0	NaN	NaN

Table S3. Statistically data processing.

Index	number	solution	root	seedling	log10_solution	germination_flg	log10_solution2
count	450.000	450.000	450.000	450.000	450.000	450.000	450.000
mean	15.500	22.220	3.226	3.148	0.900	0.400	0.400
std	8.665	39.113	1.852	2.122	0.300	1.021	1.021
min	1.00	0.000	0.000	0.000	0.000	-1.000	-1.000

Index	number	solution	root	seedling	log10_solution	germination_flg	log10_solution2
25%	8.000	0.100	2.100	2.000	1.000	0.000	0.000
50%	15.500	1.000	3.100	2.700	1.000	0.000	0.000
75%	23.000	10.000	4.500	3.800	1.000	1.000	1.000
max	30.000	100.000	9.100	9.200	1.000	2.000	2.000

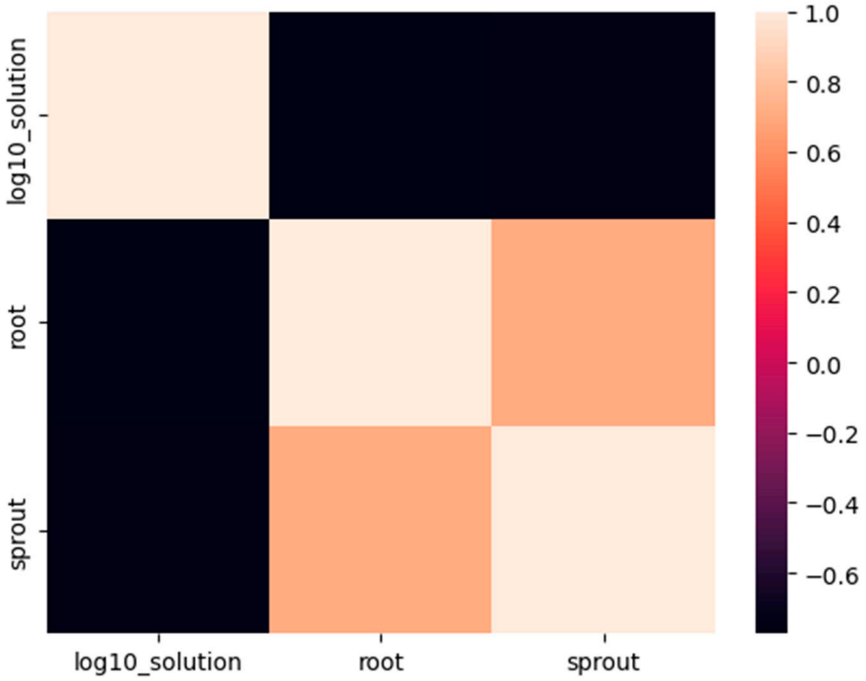

Figure S4. Correlation heat map between log of MnO_2 NPs concentration, root length and seedling (sprout) length.

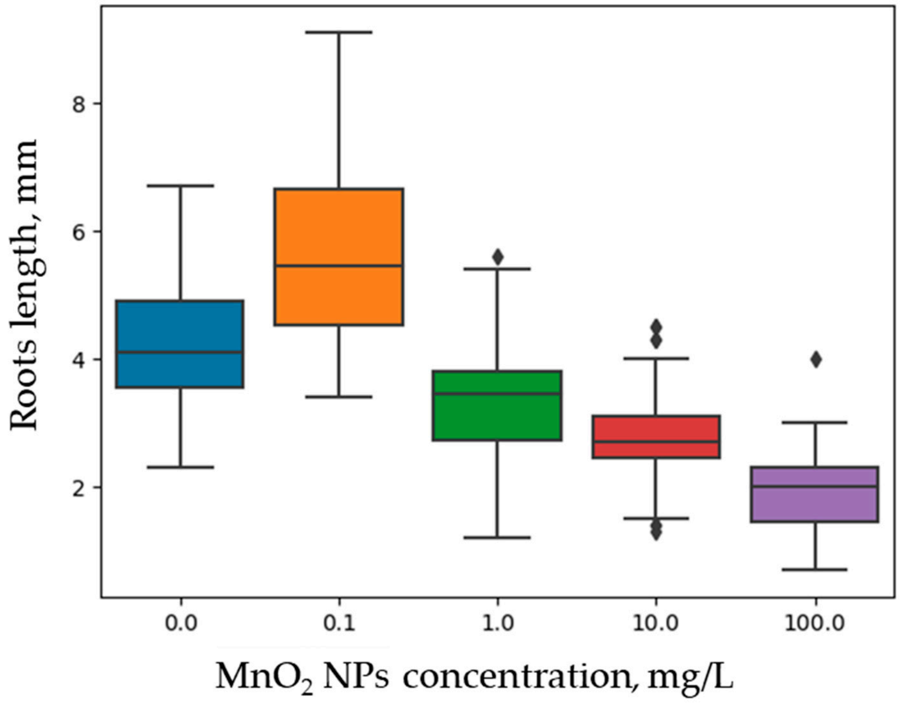

Figure S5. Box plot of dependence of roots length on MnO_2 NPs concentration.

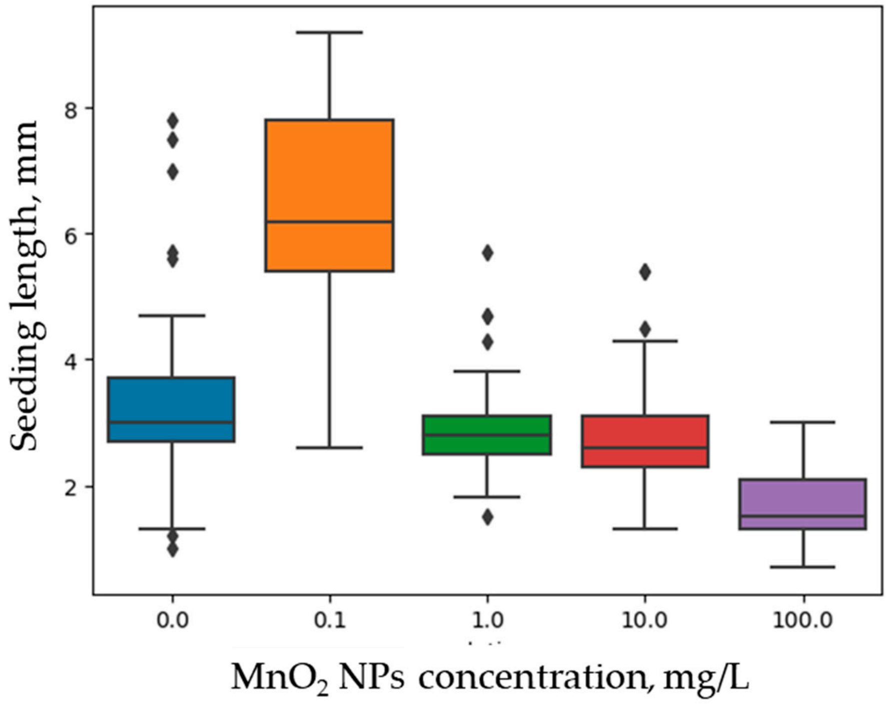

Figure S6. Box plot of dependence of seedling (sprout) length on MnO_2 NPs concentration.

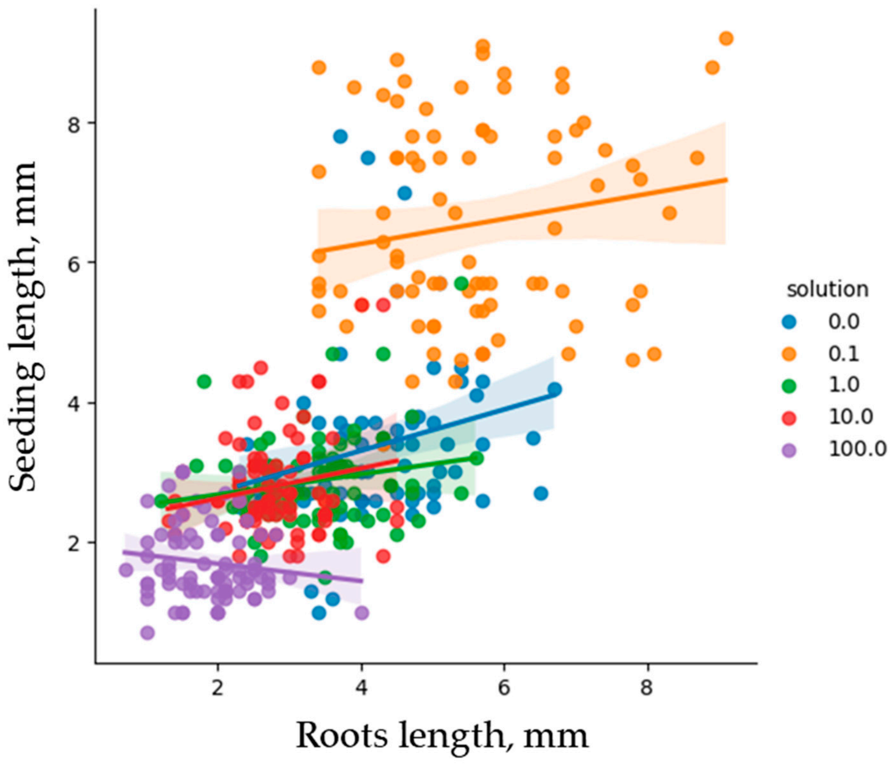

Figure S7. Distribution of length of roots and seedling.
